# Dual Feedstock Upcycling of α-Methylstyrene-Doped Poly(methyl methacrylate) and Biomass via the Telescope of Depolymerization and Diels–Alder Reaction

**DOI:** 10.1021/acs.orglett.5c00645

**Published:** 2025-04-02

**Authors:** Rui Zhang, Mason T. Chin, Tianning Diao

**Affiliations:** Department of Chemistry, New York University, 100 Washington Square East, New York, New York 10003, United States

## Abstract

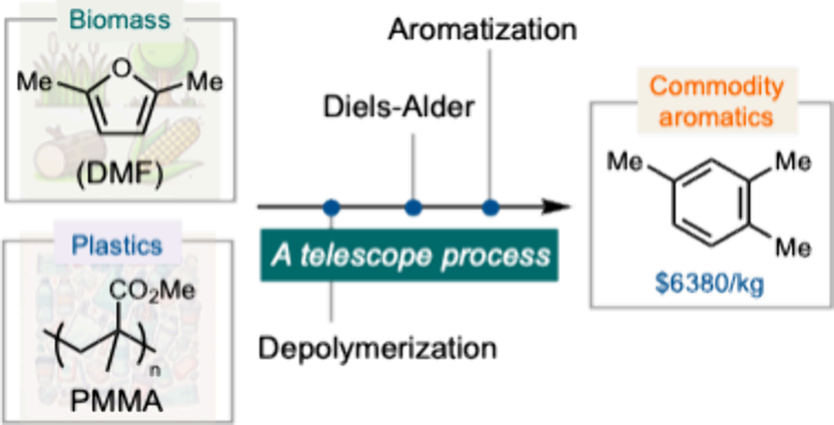

Nearly 90% of poly(methyl methacrylate) (PMMA) is not recycled and instead ends up in landfills. Conventional pyrolysis of PMMA recovers impure methyl methacrylate (MMA) with low economic value. Here, we present a telescoped dual upcycling strategy that integrates PMMA depolymerization, Diels–Alder cycloaddition, and aromatization to convert AMS-doped PMMA and biomass-derived 2,5-dimethylfuran (DMF) into 1,2,4-trimethylbenzene (pseudocumene), a valuable chemical feedstock. BBr_3_ proved effective in promoting the challenging Diels–Alder reaction between MMA and DMF under high pressure of argon.

Poly(methyl methacrylate) (PMMA) is an economical, lightweight, and shatterproof alterative to glass, widely used in window profiles, vehicle windshields, LCD screens, and monitors.^1^ Currently, nearly 90% of postconsumer PMMA waste ends up in landfills.^2,3^ PMMA is produced from methyl methacrylate (MMA) through radical polymerization at temperatures of 50–90 °C (Scheme 1).^4^ This reversible process can be altered to favor depolymerization at higher temperatures.^5^ Pyrolysis protocols at temperatures above 400 °C can crack PMMA waste and recover MMA via distillation.^6^ However, MMA recovered through this process typically contains impurities due to undesirable side reactions at high temperatures, which interfere with the circular utility of the monomer.^7^ Recent advancements in PMMA upcycling have focused on lowering depolymerization temperatures by incorporating functional groups with low bond dissociation energies (BDE).^8−10^ In this context, we reported that doping PMMA with a small amount of α-methylstyrene (AMS) enables depolymerization at 150–180 °C, allowing for the recovery of MMA in high-yield and high-purity, while preserving the mechanical strength and optical clarity of PMMA crucial for its commercial applications.^11^

**Scheme 1:**
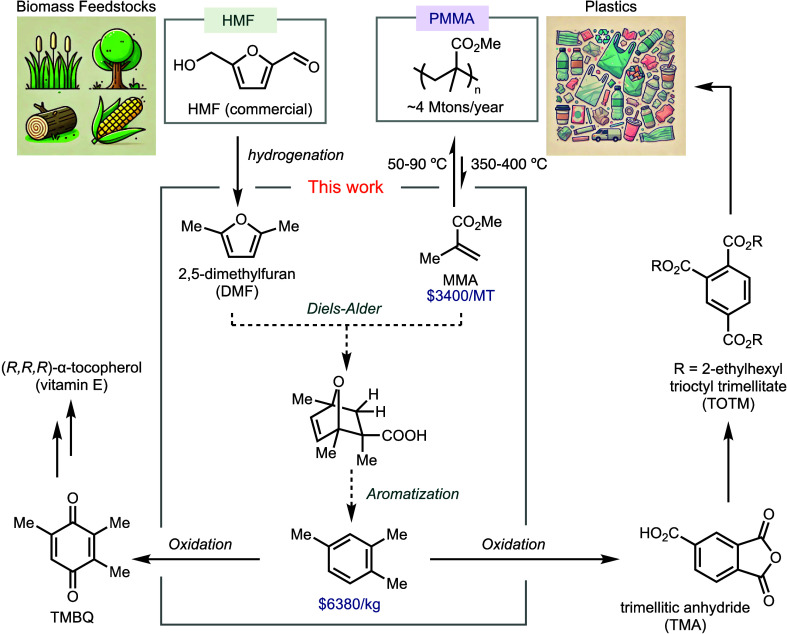
Telescoping Depolymerization/Diels–Alder Process to Convert PMMA and Cellulosic Biomass Products into Commodity Aromatics

While a circular economy offers significant promise, MMA recovered from depolymerization, even at high purity, has relatively low economic value. This limitation often fails to justify the energy, effort, and costs required for its recovery. A more compelling alternative is to upcycle PMMA into higher-value chemical feedstocks that serve as precursors for the synthesis of high-value products and materials. This approach could improve the economic feasibility and appeal of PMMA recycling on a practical scale.

Biomass represents a sustainable chemical feedstock for replacing fossil resources.^12^ Recognizing this potential, extensive research has focused on transforming sugars from cellulosic biomass, leading to commercial processes for producing furfural and hydroxymethylfurfural (HMF).^13^ The reduction of HMF, either through hydrogenation or electrocatalytic reduction, yields 2,5-dimethylfuran (DMF), a promising fuel with high-octane ratings (indicating high resistance to explosion), high energy density, and low water solubility.^14,15^

We hypothesize that a Diels–Alder reaction between MMA and DMF could drive the depolymerization of PMMA by irreversibly converting MMA following the Le Chatelier’s principle (Scheme 1). Herein, we report an initial investigation of this concept, demonstrating a telescoped depolymerization of AMS-doped PMMA followed by a Diels–Alder reaction with DMF, leading to aromatization and the formation of 1,2,4-trimethylbenzene (pseudocumene). Traditionally obtained through oil refining, 1,2,4-trimethylbenzene is a valuable chemical feedstock used in the production of both commodity materials and fine chemicals, such as vitamin E^16^ and trioctyl trimellitate (TOTM), a plasticizer that enhances the flexibility and durability of poly(vinyl chloride) (PVC).^17^ This approach not only addresses challenges in PMMA upcycling but also facilitates the conversion of biomass, a renewable chemical feedstock, into petroleum-derived products through more environmentally favorable plastic waste streams.

The Diels–Alder reaction of furfuryl derivatives has been explored with various dienophiles (Scheme 2).^18^ However, using MMA as a dienophile presents significant challenges.^19^ The 1,1-donor–acceptor substituents of MMA electronically and sterically reduce its reactivity, often necessitating the use of highly activated dienes.^20−22^ Moreover, DMF is also a relatively unreactive diene due to steric hindrance. Diels–Alder reactions involving DMF as the diene typically require sterically accessible alkenes, such as ethylene^23^ or acrylic acid.^24−26^ Diels–Alder reactions between DMF and 1,1-disubstituted alkenes remain an unmet challenge, as these reactions often require a large excess of DMF and result in low yields.^27−29^

**Scheme 2:**
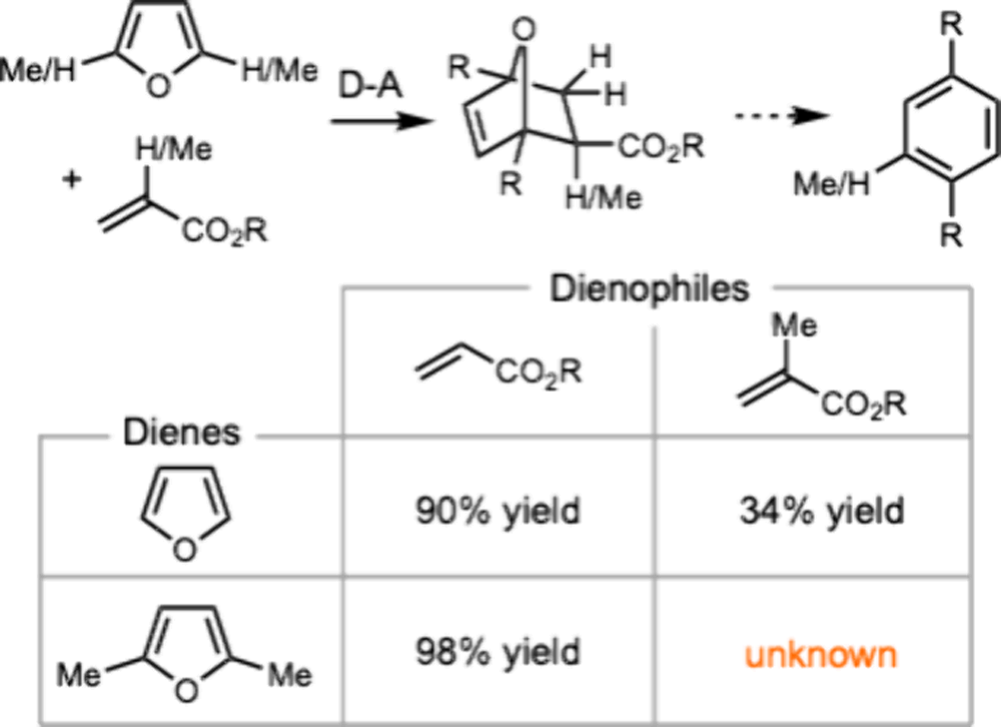
Precedents and Limitations of Diels–Alder Reactions between Acrylate Esters and Furans

We began our study by developing the conditions for the Diels–Alder reaction between MMA **1** and DMF **2** (Table 1). Heating MMA **1** and DMF **2** in the presence of a heterogeneous Brønsted acid Bi-BTC (BTC = benzenetricarboxylic acid)^25^ resulted in no conversion to either the Diels–Alder adduct or the aromatization product **3** (entry 1). When we conducted the reaction with the strong Brønsted acidic ionic liquid [Bmim]HSO_4_^24^ at 120 °C, we observed the formation of **3** in 17% yield (entry 2). However, the reaction reproducibility was poor, with yields varying across different batches of [Bmim]HSO_4_. Screening a range of Lewis acids (entries 3–12) identified BBr_3_ as particularly effective, affording **3** in 59% yield along with 18% of the benzyl bromide **4** as a mixture of regioisomers (entry 12). Benzylic bromination with BBr_3_ has been reported to proceed via hydride abstraction by the *in situ* formed [BBr_2_]^+^[BBr_4_]^−^.^30^ With 1.5 equiv of BBr_3_, adding Na_2_SO_4_ as a water scavenger (entry 13) and applying 8–10 atm of argon pressure further promoted the Diels–Alder reaction (entry 14). In particular, conducting the reaction under high pressure enabled us to lower the Diels–Alder reaction temperature to room temperature, which minimized byproduct formation and substrate decomposition while maintaining high conversion.^27^ Further heating the mixture to 80 °C promoted decarboxylation and aromatization, affording **3** in 83% yield (entry 14).

**Table 1:**
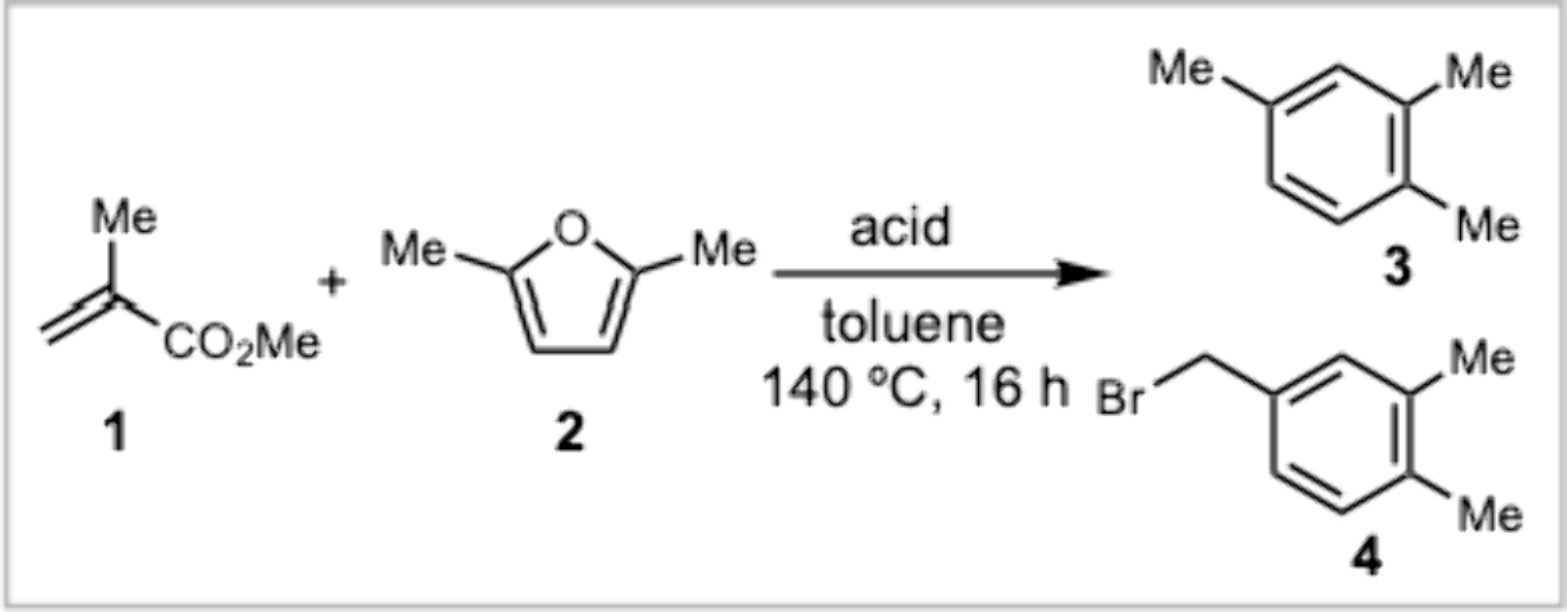
Condition Development for the Diels–Alder Reaction between MMA and DMF^a^

Entry	Acid	Yield of **3** (%)	Yield of **4** (%)
1	Bi-BTC	ND	ND
2^b^	[Bmim]HSO_4_	17	ND
3	BF_3_-Et_2_O	ND	ND
4	B(C_6_F_5_)_3_	ND	ND
5	AlCl_3_	ND	ND
6	ZnCl_2_	ND	ND
7	SnCl_4_	5	ND
8	In(OTf)_3_	ND	ND
9	Sc(OTf)_3_	ND	ND
10	Eu(OTf)_3_	ND	ND
11^c^	BCl_3_	3	3
12	BBr_3_	59	18
13^d^	BBr_3_	64	17
14^d^^,^^e^	BBr_3_	83	2

a Reaction conditions: **1** (0.20 mmol), **2** (0.30 mmol), acid (0.30 mmol), toluene (1.0 mL) at 140 °C, 16 h. Yields determined by GC-MS.

b [Bmim]HSO_4_ = 1-butyl-3-methylimidazolium hydrogen sulfate (0.80 mmol), 120 °C.

c BCl_3_ (0.40 mmol), byproduct is chlorinated arene instead of **4**.

d Na_2_SO_4_ (100 mg), 0.05 M.

e Under 8–10 atm of argon at room temperature for 16 h then at 80 °C for an additional 12 h. ND = not detected.

Subsequently, we applied the optimized Diels–Alder conditions to a telescoped sequence involving the depolymerization of PMMA, Diels–Alder cyclization, and aromatization, enabling the conversion of PMMA and DMF into aromatic products (Scheme 3). We selected AMS-doped PMMA P(MMA-*co*-AMS) as a technical PMMA plastic to demonstrate this strategy.^11^ We first heated P(MMA-*co*-Nap) (Nap = 2-naphthyl) to 210 °C in the presence of monomethyl ether of hydroquinone (MEHQ) to induce depolymerization while preventing MMA repolymerization. The generated MMA was condensed in a receiving flask containing DMF. After the addition of BBr_3_ via vacuum transfer and the condensation of argon (8–10 atm), the mixture was stirred at room temperature for 16 h, then heated to 80 °C for an additional 12 h, affording **3** in 55% yield over two steps, with a minor fraction converted to **4**. Applying the same protocol to P(MMA-*co*-Anthr) (Anthr = 2-anthracenyl) under depolymerization conditions at 190 °C yielded **3** in 67% yield, along with 2% of **4**.

**Scheme 3:**
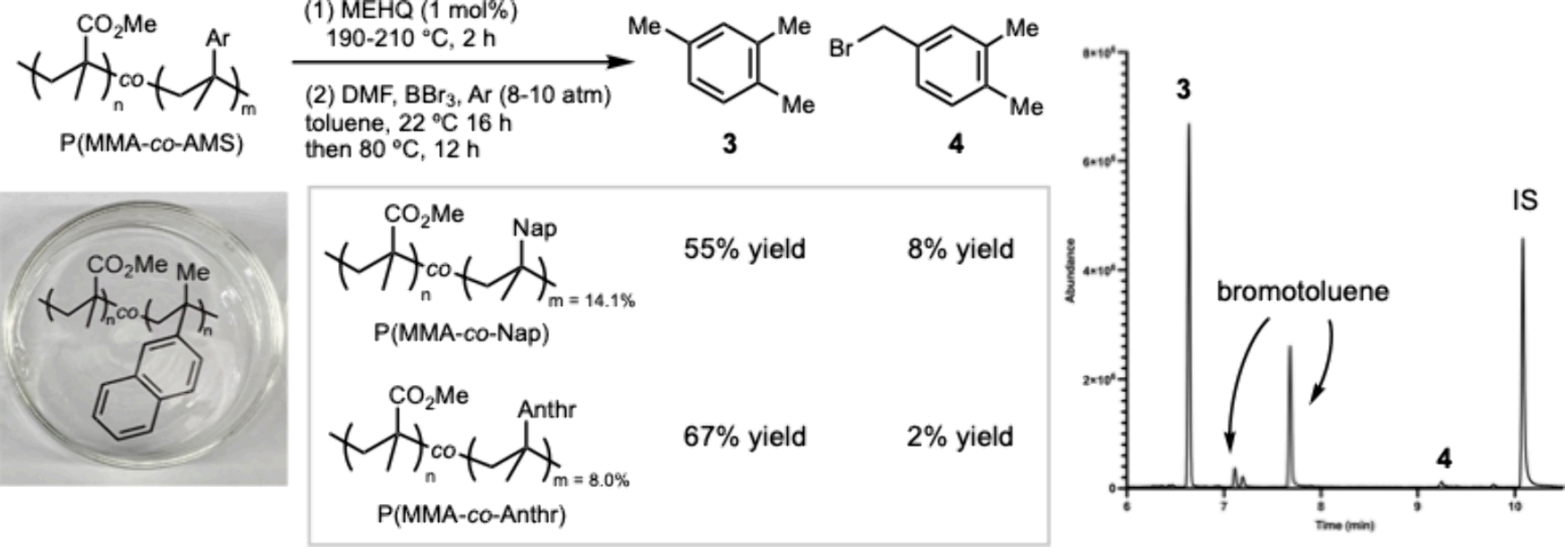
Conversion of PMMA to Aromatic Products via a Telescoping Protocol

We conducted experiments to investigate potential Diels–Alder cycloaddition intermediates before aromatization and to elucidate the role of BBr_3_ in promoting the reaction. Performing the Diels–Alder reaction between **1** and **2** with an excess of **1** and a reduced loading of BBr_3_ enabled us to observe the formation of intermediate **6**, detected by GC-MS as a mixture of the *endo* and *exo* diastereomers in a ratio of 1:1.7 (Scheme 4). Using flash column chromatograph, we isolated a small amount of **6-*****exo***, whose connectivity and stereochemistry were confirmed by HSQC and HMBC (Figures S16–S17).

**Scheme 4:**
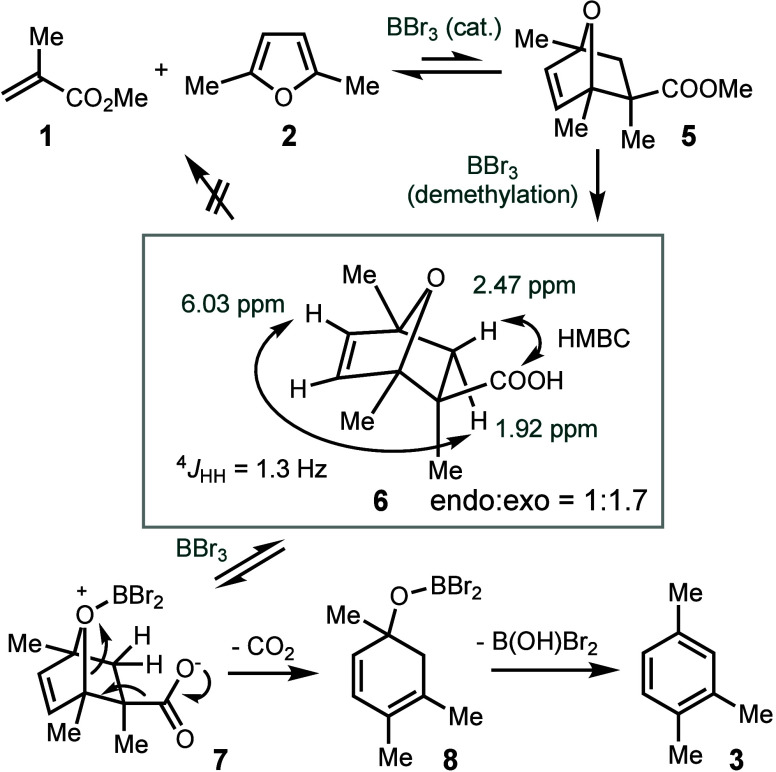
Isolation of Diels–Alder Addition Product and Proposed Mechanism of BBr_3_-Catalyzed Diels–Alder/Aromatization of MMA and DMF

The isolation of intermediate **6** led us to propose that BBr_3_ catalyzes the Diels–Alder reaction between MMA and DMF, forming intermediate **5** (Scheme 4). However, we did not observe **5** as an intermediate, suggesting that the equilibrium favors the retro-Diels–Alder, likely due to the aromaticity of DMF and the steric hindrance of both substrates. This explains why most Lewis acid catalysts are ineffective. BBr_3_, on the other hand, promotes this reaction not only by acting as a Lewis acid but also by serving as a demethylation reagent, converting ester **5** into carboxylic acid **6**,^31^ an irreversible process that drives the Diels–Alder reaction equilibrium forward. The high pressure of argon facilitates the formation of a compact transition state, promotes cycloaddition because the product occupies a smaller volume compared to the reactants, and increases the effective concentration of the reactants. Subsequently, BBr_3_ catalyzes the decarboxylation of **6** through coordination, followed by elimination to generate **3**.

In summary, we demonstrate a telescoped protocol that integrates depolymerization, Diels–Alder cycloaddition, and aromatization to convert PMMA and biomass-derived DMF into a valuable commodity aromatic product, 1,2,4-trimethylbenzene. The use of BBr_3_ and high-pressure argon overcomes the challenges associated with the Diels–Alder reaction of sterically bulky, aromatic dienes and bulky dienophiles. Although challenges remain in applying this strategy to the practical recycling of PMMA waste, this proof-of-concept study paves the way for the dual upcycling of plastic waste and biomass into high-value aromatic chemicals, offering a sustainable alternative to petroleum-sourced commodity production. Future optimization efforts will focus on rendering the reaction catalytic and reducing the cost of the reagents.

## Data Availability

The data underlying this study are available in the published article and its Supporting Information.

## Supporting Information Available

Full experimental procedures, characterization data, and NMR spectra (PDF)
