# Supplementary material for: Dual Feedstock Upcycling of α-Methylstyrene-Doped Poly(methyl methacrylate) and Biomass via the Telescope of Depolymerization and Diels–Alder Reaction
Source: Org Lett. 2025 Apr 2;27(14):3590–4. doi: 10.1021/acs.orglett.5c00645 (PMC11998077; doi:10.1021/acs.orglett.5c00645)
Supplement: Supplementary file 1 — ol5c00645_si_001.pdf [file ol5c00645_si_001.pdf]

Supporting information

Dual Feedstock Upcycling of α-Methylstyrene-Doped Poly(methyl methacrylate) and Biomass via the Telescope of Depolymerization and Diels-Alder Reaction

Rui Zhang, Mason Chin, and Tianning Diao*

Department of Chemistry, New York University, 100 Washington Square East, New York, NY 10003, United States

*E-Mail: diao@nyu.edu

Table of contents

1. General comments S2

2. General procedure for the optimization of Diels–Alder reaction between MMA and DMFS3

3. Optimized procedure for Diels–Alder reaction S6

4. Optimized procedure for one-pot depolymerization and Diels–Alder reaction S8

4.1 Synthesis of copolymers P(MMA-*co*-Nap) and P(MMA-*co*-Ant) S8

4.2 Optimized argon-condensed procedure for one-pot depolymerization and Diels–Alder reaction S9

5. Diels-Alder intermediates S13

1. General comments

Common solvents (e.g., toluene) were dried and deoxygenated by passing through alumina in a solvent purification system. Deuterated solvents were purchased from commercial sources. Unless otherwise specified, reagents were purchased from commercial sources.

Compounds were prepared according to the literature procedure.[1] MMA was purchased and distilled before use (bp = 98-100 ºC). BBr_3 in DCM was purchased and used without purification. BBr_3 decomposes in air and was stored in desiccator under 2-8 °C after open.

NMR spectra were recorded on a Bruker Avance 400 spectrometer (400 MHz for ^1H, and 101 MHz for ^{13}C) or a Bruker Avance 500 spectrometer (500 MHz for ^1H and 126 MHz for ^{13}C). Unless otherwise noted, ^1H and ^{13}C chemical shifts are reported relative to tetramethylsilane (TMS) in ppm at room temperature. ^1H chemical shifts are referenced to residual proteo-solvent peak at 7.26 ppm ($CHCl_3$), 2.05 ppm ($(CD_2H)C(O)(CD_3)$); ^{13}C chemical shifts are referenced to 77.1 ppm ($CDCl_3$), 29.8 ppm ($(CD_3)_2CO$). Structural assignments were made with additional information from gHSQC and gHMBC experiments.

Gel-permeation chromatography (GPC) was carried out using a Shimadzu pump coupled to a Shimadzu RI detector controlled by an EZStart program. M_n^{app} and dispersity (*Đ*) represent the apparent number-average molecular weight and dispersity index respectively.

High resolution mass spectra (HRMS) were recorded on an Agilent 6224 TOF LC/MS (APCI source).

GC-MS data was obtained using a Shimadzu GCMS-TQ8040 with a Shimadzu SH-Rxi-5Sil MS column (L 30 m, ID 0.25 mm, DF 0.25 μm). For 1,2,4-trimethylbenzene (P, product), t_R = 6.63 min; For 1,3,5-trimethoxylbenzene (IS, internal standard), t_R = 10.07 min.

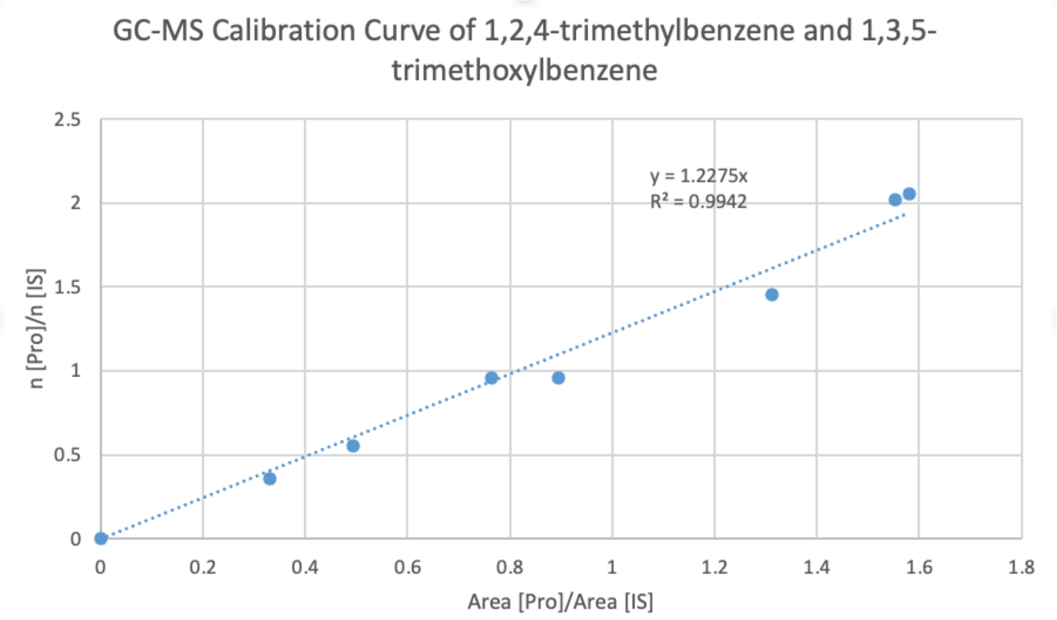

Figure S1. GC-MS Calibration Curve of 1,2,4-Trimethylbenzene and 1,3,5-Trimethoxylbenzene.

---

1. Chin, M. T.; Yang, T.; Quirion, K. P.; Lian, C.; Liu, P.; He, J.; Diao, T., Implementing a Doping Approach for Poly(methyl methacrylate) Recycling in a Circular Economy. *J. Am. Chem. Soc.* **2024,** *146*, 5786-5792.

2. General procedure for the optimization of Diels–Alder reaction between MMA and DMF

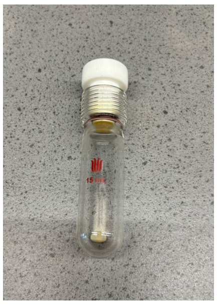

Figure S2. Thick-wall pressure-tube for the Diels–Alder reaction.

Under N_2, an oven-dried pressure-tube equipped with a magnetic stir bar was charged with methyl methacrylate (MMA, 20.0 mg, 21.2 µL, 0.20 mmol, 1.0 equiv.), 2,5-dimethylfuran (DMF, 28.8 mg, 31.9 µL, 0.30 mmol, 1.5 equiv.), Na_2SO_4 (100 mg), toluene (4.0 mL), followed by addition of boron tribromide (BBr_3, 1.0 M in DCM, 0.30 mL, 1.5 equiv.). The reaction was immediately placed to a stir plate, which has been preheated to the desired temperature. The reaction was stirred at 600 rpm for 16 hours. Upon completion, the reaction mixture was cooled to room temperature and quenched with water (3 mL). The mixture was extracted with ethyl acetate (5 mL x 3) and the combined organic phases were analyzed using quantitative GC-MS, with 1,3,5-trimethoxybenzene as an internal standard.

Table S1 Effect of Boron Reagent[a]

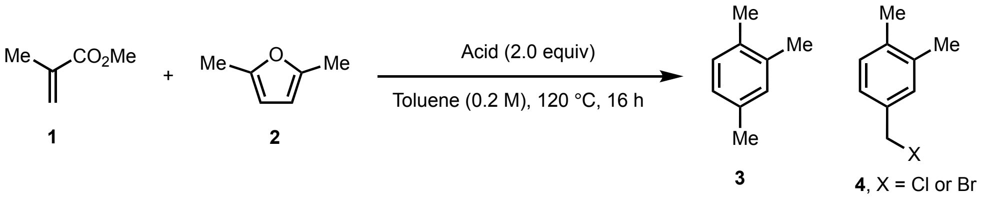

Entry	Acid	Yield of 3 (%)[b]	Yield of 4 (%)[c]
1[d]	[Bmim]HSO_4	17	ND
**2**	**BBr_3**	**7**	**17**
3	BCl_3	3	3
4	BF_3•Et_2O	ND	ND
5	BF_3•THF	ND	ND
6	$B(C_6F_5)_3$	ND	ND

[a] Unless otherwise noted, all reactions were performed in toluene (1.0 mL) at 120 °C for 16 h with MMA (0.20 mmol), 2,5-dimethylfuran (0.30 mmol), in the presence of acid (0.40 mmol). ND = not detected. [b] Determined by GC-MS. [c] Determined by GC-MS with assuming that the response factor of all products is same. [d] [Bmim]HSO_4 = 1-butyl-3-methylimidazolium hydrogen sulfate, 4.0 equiv., 120 ºC.

Table S2 Effect of the Amounts of MMA and BBr_3[a]

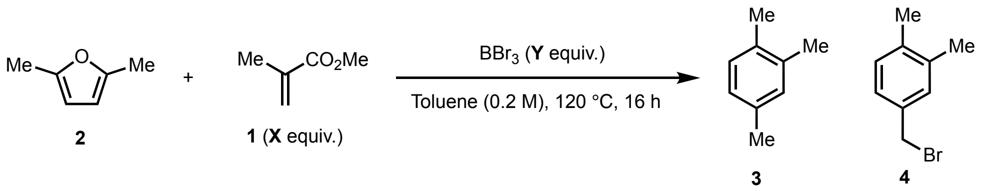

Entry	X	Y	Yield of 3 (%)[b]	Yield of 4 (%)[c]
1	1.5	0.5	41	6
**2**	**1.5**	**1.0**	**52**	**14**
3	1.5	2.0	12	43
4	2.0	1.0	32	9
5	3.0	1.0	38	6
6	2.0	2.0	14	29
7	5.0	2.0	18	8
8	5.0	2.0	13	8
9	5.0	5.0	19	9

[a] Unless otherwise noted, all reactions were performed in toluene (1.0 mL) at 120 °C for 16 h with 2,5-dimethylfuran (0.20 mmol), MMA, in the presence of BBr_3. ND = not detected.[b] Determined by GC-MS. [c] Determined by GC-MS with assuming that the response factor of all products is same.

Table S3 Effect of the Solvent and Temperature[a]

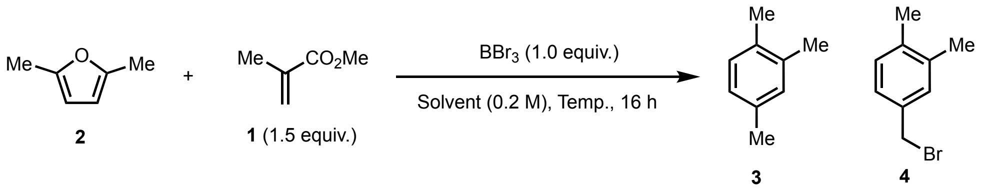

Entry	Solvent	Temperature (°C)	Yield of 3 (%)[b]	Yield of 4 (%)[c]
1[d]	Toluene	100	24	9
2	Toluene	120	52	14
3	Toluene	140	60	15
**4[e]**	**Toluene**	**140**	**59**	**18**
5	Toluene	160	57	11
6	*N*,*N*-Dimethylformamide	140	ND	ND

7	DMSO	140	ND	ND
8	DCE	140	9	ND

[a] Unless otherwise noted, all reactions were performed for 16 h with 2,5-dimethylfuran (0.20 mmol), MMA (0.30 mmol), in the presence of BBr_3 (0.20 mmol). ND = not detected. [b] Determined by GC-MS. [c] Determined by GC-MS with assuming that the response factor of all products is same. [d] Proceeded 24 hours. [e] MMA (0.20 mmol), 2,5-dimethylfuran (0.30 mmol) in the presence of BBr_3 (0.30 mmol).

Table S4. Effect of other Lewis Acids[a]

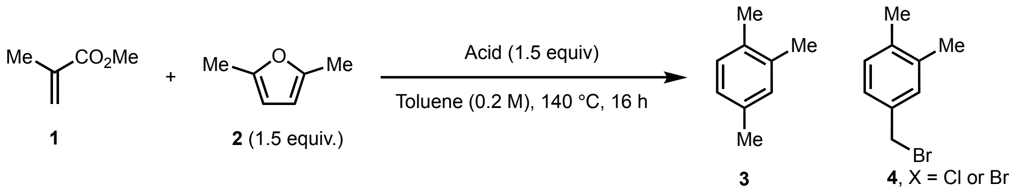

Entry	Lewis acid	Yield of 3(%)[b]	Yield of 4 (%)[c]
1	BBr_3	59	18
2	BCl_3	ND	ND
3	BF_3-Et_2O	ND	ND
4	$B(C_6F_5)_3$	ND	ND
5	Bi-BTC	ND	ND
5	$AlCl_3$	ND	ND
6	$ZnCl_2$	ND	ND
7	$SnCl_4$	5	ND
8	$In(OTf)_3$	ND	ND
9	$Sc(OTf)_3$	ND	ND
10	$Eu(OTf)_3$	ND	ND
**11**[d]	**BBr_3**	**64**	**17**

[a] Unless otherwise noted, all reactions were performed in toluene (1.0 mL) at 140 °C for 16 h with 2,5-dimethylfuran (0.30 mmol), MMA (0.20 mmol). ND = not detected. [b] Determined by GC-MS. [c] Determined by GC-MS with assuming that the response factor of all products is same. [d] Na_2SO_4 (100 mg) was using as the additive, the concentration is 0.05 M.

3. Optimized procedure for Diels–Alder reaction

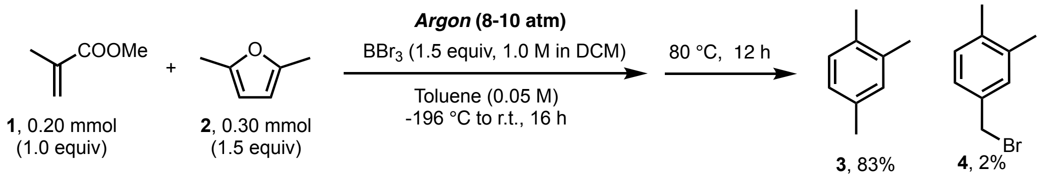

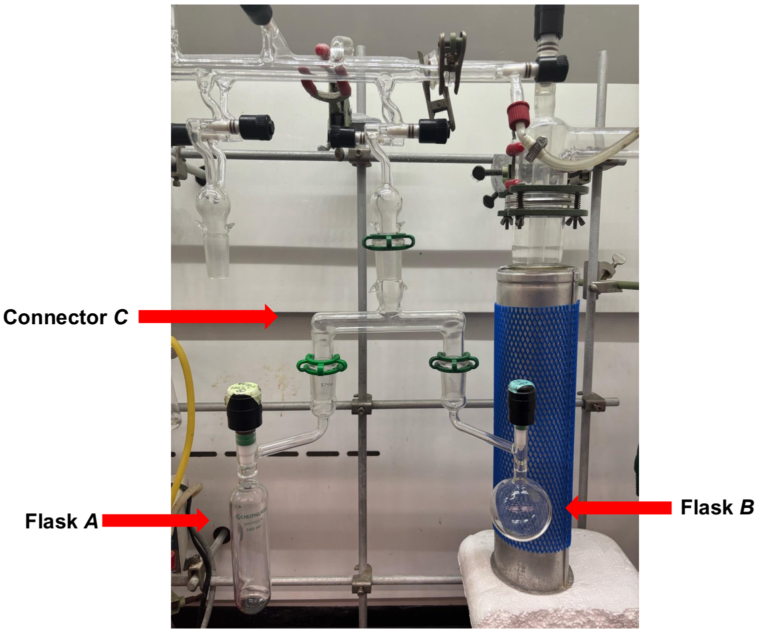

Figure S3. Experimental setup for the Diels-Alder reaction under 8-10 atm of argon.

All glassware was dried overnight in an oven prior to use. A sealed 100 mL Schlenk flask (Flask *A*) containing a magnetic stir bar was charged with methyl methacrylate (MMA, 20.0 mg, 21.2 µL, 0.20 mmol, 1.0 equiv.), 2,5-dimethylfuran (28.8 mg, 31.9 µL, 0.30 mmol, 1.5 equiv.), and toluene (4.0 mL). Another Schlenk flask (Flask *B*) was charged with boron tribromide (BBr_3, 1.0 M in DCM, 0.30 mL, 1.5 equiv.) and a freeze-pump-thaw cycle was performed for both Flask *A* and Flask *B*. Then BBr_3 was introduced to Flask *A* via vacuum-transfer. After the transfer, Flask *A* was slowly opened to allow argon to condense in the liquid nitrogen-cooled flask, ensuring that the condensed volume was roughly 1/100th of that in Flask *A*. Flask *A* was then removed from the liquid nitrogen and stirred at room temperature for 16 hours. When Flask *A* is removed from the liquid nitrogen, the argon inside will vaporize to create an argon atmosphere at 8-10 atm during the reaction. Subsequently, Flask *A* was placed in an oil bath at 80 °C and stirred for an additional 12 hours. The reaction was cooled to room temperature and quenched with water (3 mL) and extracted with ethyl acetate (5 mL x 3). After addition of 1,3,5-trimethoxybenzene as the internal standard, the sample was analyzed using GC-MS, producing an average yield of 83% for 1,2,4-trimethylbenzene, based on results from three identical experiments.

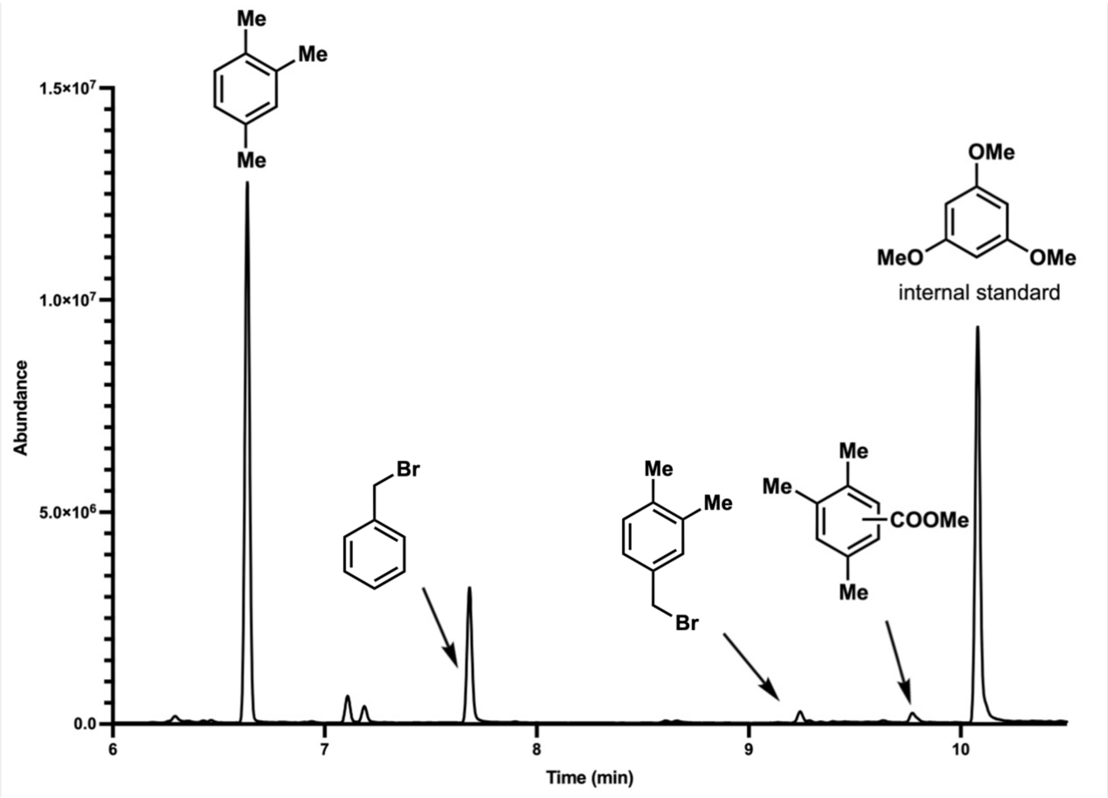

Figure S4. GC-MS spectrum of Diels–Alder reaction under 8-10 atm of argon.

4. Optimized procedure for one-pot depolymerization and Diels–Alder reaction

4.1 Synthesis of copolymers P(MMA-*co*-Nap) and P(MMA-*co*-Ant)

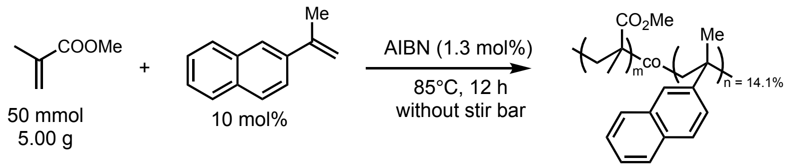

In a flame-dried round-bottom flask, AIBN (106.7 mg, 0.65 mmol, 0.013 equiv.) and 2-isopropenylnaphthalene (841 mg, 5.0 mmol, 0.10 equiv.) were added under a nitrogen atmosphere. Freshly distilled methyl methacrylate (MMA, 5.00 g, 50.0 mmol, 1.0 equiv.) was introduced via syringe transfer, and the reaction mixture was heated at 85 °C for 16 h. After cooling to room temperature, the reaction mixture was dissolved in dichloromethane (DCM). The resulting solution was then layered with methanol, and the precipitate was collected by centrifugation, and the supernatant was discarded. This precipitation process was repeated twice and the resulting product was dried under vacuum for 3 h to yield the purified polymer as white solid, with a weight of 2.73 g. The incorporation of the comonomer (n = 14.1%) was determined by ^{1}H NMR in acetone-d_6. The peaks ranging from 3.64 ppm to 3.47 ppm are attributed to the methoxy group (COOMe), while the aromatic region peaks correspond to naphthalene.

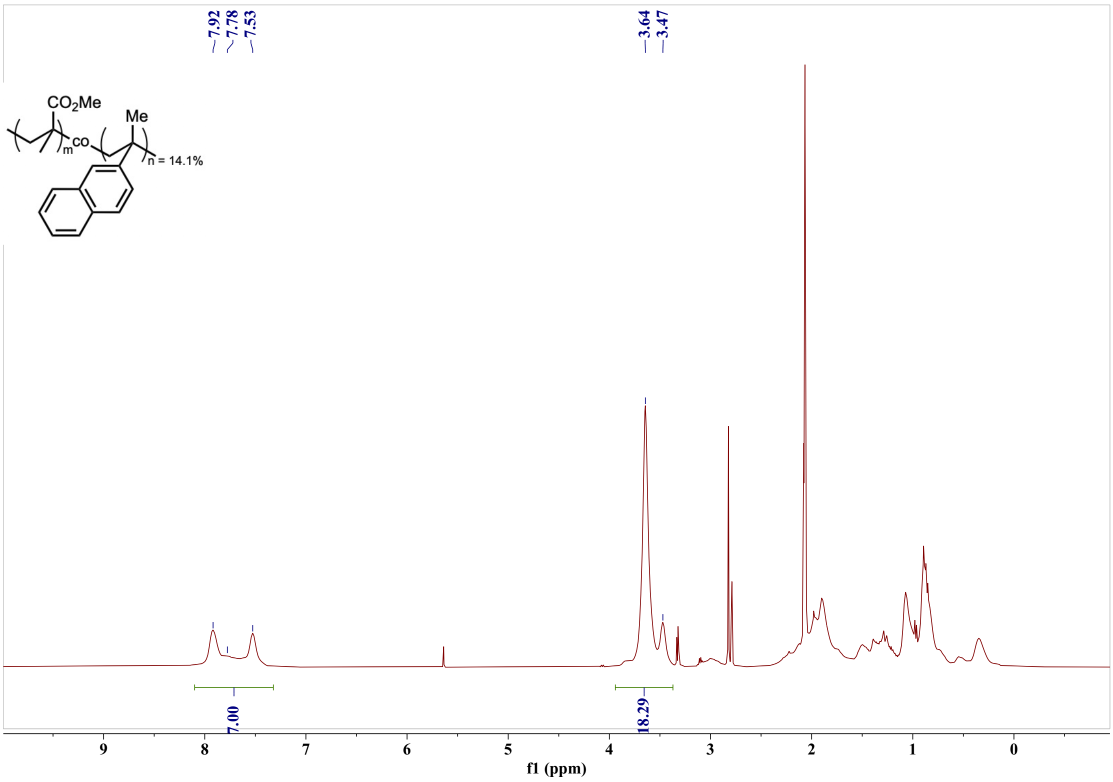

Figure S5. ^{1}H-NMR (400 MHz, acetone-*d6*) spectrum of P(MMA-*co*-Nap). ($M_w = 1.68 \times 10^4$ Da, $M_n = 7.21 \times 10^3$ Da, $Đ = 2.33$)

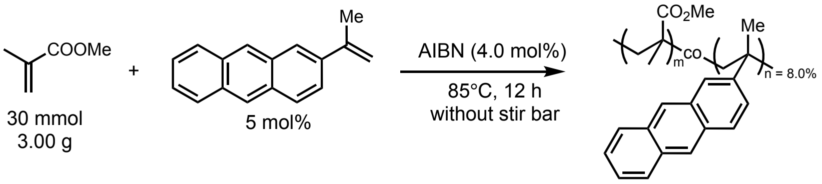

Followed the procedure described above, AIBN (197.1 mg, 0.40 mmol, 0.040 equiv.), 2-isopropenylanthracene (328 mg, 1.50 mmol, 0.050 equiv.) and MMA (freshly distilled, 3.00 g, 30.0 mmol, 1.0 equiv.) were used. The final product yielded 910 mg of a light yellow solid. The incorporation of the comonomer (n = 8.0%) was determined by ^{1}H NMR in acetone-d_6. The peaks ranging from 3.93 ppm to 3.44 ppm are attributed to the methoxy group (COOMe), while the aromatic region peaks correspond to anthracene. The corresponding thermal stability data for AMS-doped PMMA could be found in literature[1].

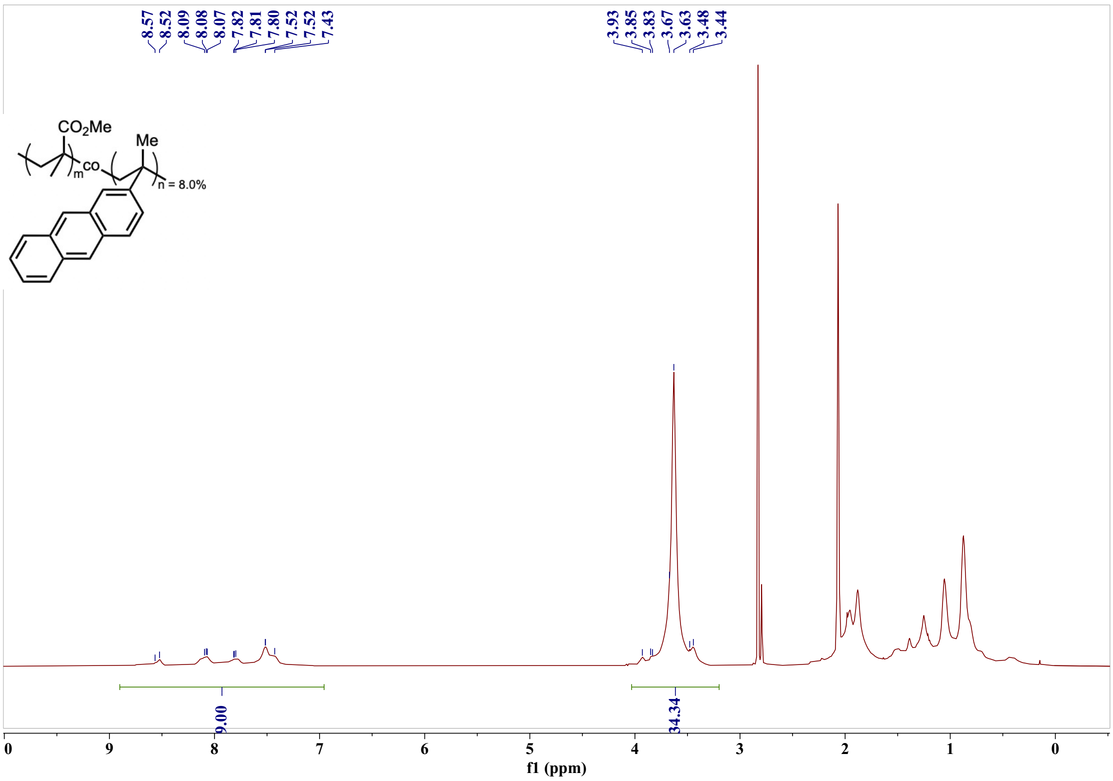

Figure S6. ^{1}H-NMR (400 MHz, acetone-*d6*) spectrum of P(MMA-*co*-Ant). ($M_w = 3.70 \times 10^3$ Da, $M_n = 2.65 \times 10^3$ Da, $Đ = 1.39$)

4.2 Optimized argon-condensed procedure for one-pot depolymerization and Diels–Alder reaction

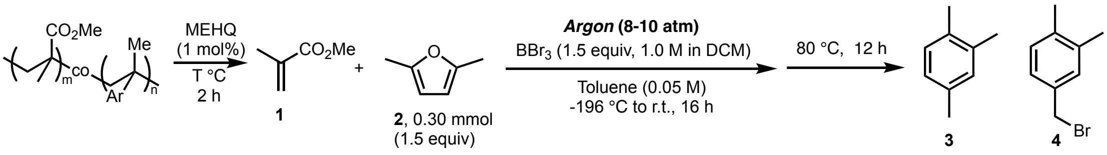

Entry	Ar	n (%)	T (°C)	Yield of **3** (%)	Yield of **4** (%)
1	2-naphthyl	14.1	210	55	8
2	2-anthryl	8.0	190	67	2

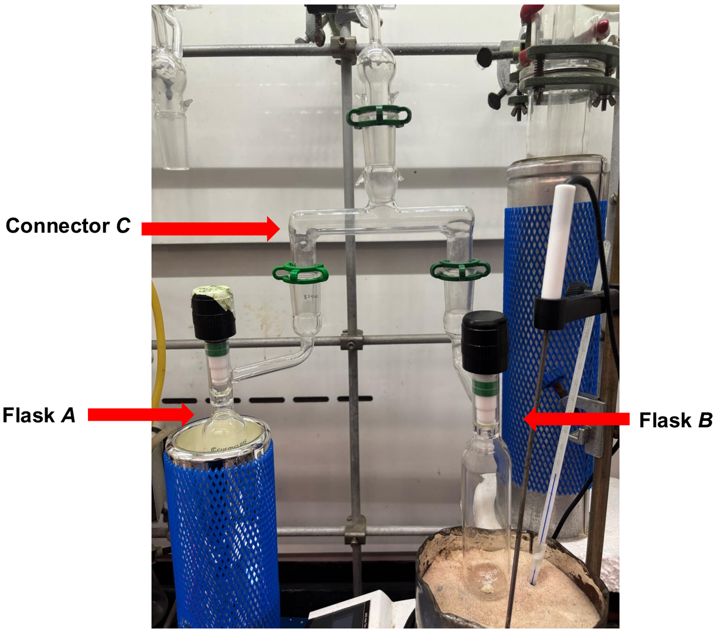

Figure S7. Experimental setup for the one-pot depolymerization and Diels–Alder reaction under 8-10 atm of argon (depolymerization part).

All glassware was dried overnight in an oven before use. A solution of 2,5-dimethylfuran (28.8 mg, 0.30 mmol, 1.5 equiv.) in toluene (4 mL) was added to Flask *A*, which was equipped with a magnetic stirrer. Copolymer containing 0.20 mmol of MMA (25.5 mg for P(MMA-*co*-Nap) and 23.8 mg for P(MMA-*co*-Ant)) was combined with 4-methoxyphenol (MEHQ, 0.0020 mmol, 10 µL of a 0.20 M solution in toluene) in Flask *B*. A freeze-pump-thaw cycle was performed on Flask *A*, reaching a vacuum of 70 millitorr (~9.3 Pa) and immersed in liquid nitrogen. Flask *B* was evacuated and placed in a preheated sand bath at 190 °C for P(MMA-*co*-Ant) or 210 °C for P(MMA-*co*-Nap) for 2 hours. During the reaction, the copolymer gradually dissolved, leading to the formation of a white solid in the upper section of Flask *B*. Analysis by ^{1}H NMR identified the solid as 2-isopropenylnaphthalene for Entry 1 and 2-isopropenylanthracene for Entry 2.

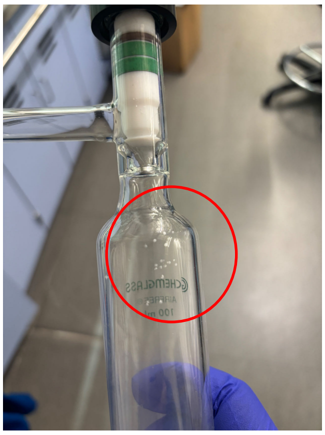

Figure S8. White solid on the top half part of Flask *B* after depolymerization.

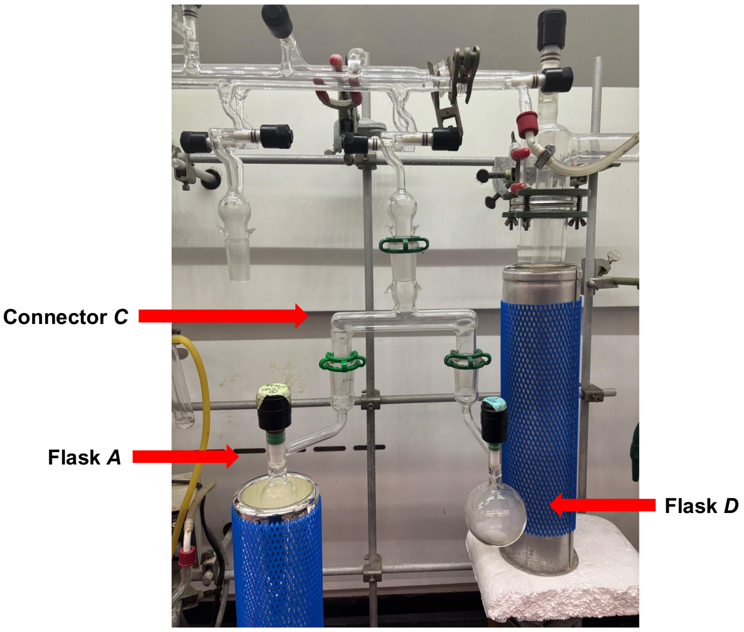

Figure S9. Experimental setup for the one-pot depolymerization and Diels–Alder reaction under 8-10 atm of argon (Diels–Alder reaction part).

After the depolymerization, boron tribromide (BBr_3, 0.3 mL, 1.0 M in DCM, in Flask *D*) was introduced to the MMA containing flask via vacuum-transfer, followed by charge of 8-10 atm argon. The reaction was warmed to room temperature and stirred for 16 hours, followed by heating to 80 °C in an oil bath for an additional 12 hours. The reaction was cooled to room temperature and quenched with water (3 mL), and extracted with ethyl acetate (5 mL x 3). After addition of 1,3,5-trimethoxybenzene as the internal standard, the sample was analyzed using GC-MS.

For the copolymer P(MMA-*co*-Nap) (Nap = 14.1%), the yield of **3** was 55%, while that of **4** was 8%. In the case of P(MMA-*co*-Ant) (Ant = 8.0%), the yield of **3** was 67%, with **4** yielding 2%.

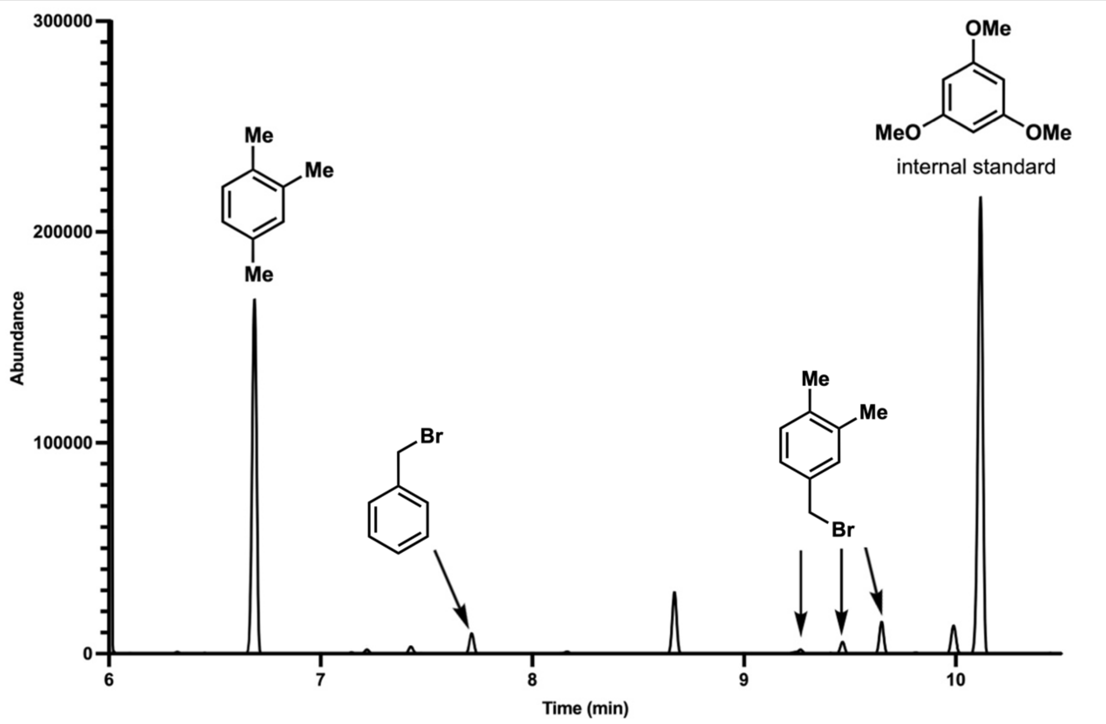

Figure S10. GC-MS spectrum of dual upcycling of P(MMA-*co*-Nap) with DMF.

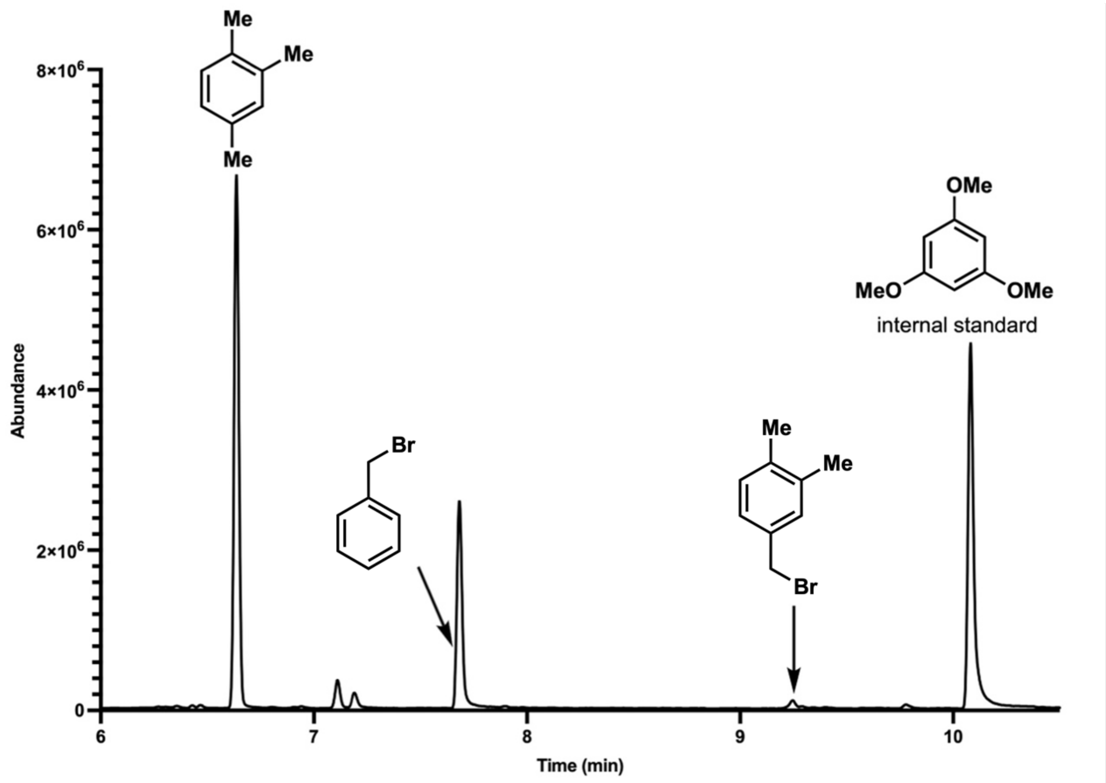

Figure S11. GC-MS spectrum of dual upcycling of P(MMA-*co*-Ant) with DMF.

5. Diels-Alder intermediates

Intermediate **6** was isolated through a Diels-Alder reaction, which is **2** (19.2 mg, 0.20 mmol, 1.0 equiv.), **1** (60.1 mg, 0.60 mmol, 3.0 equiv.), and BBr_3 (0.2 mL, 1.0 M in DCM, 1.0 equiv.) were stirred in toluene (4 mL) at 140 ºC for 16 hours. The yield was assessed via GC-MS using a pre-established calibration curve for **3**. The mass of intermediate **6** was confirmed by high-resolution mass spectrometry (HRMS). The structures of the major isomer, *exo*-**6**, were further supported by $^{1}H/^{13}C\{^{1}H\}$ heteronuclear single quantum coherence (HSQC) and $^{1}H/^{13}C\{^{1}H\}$ heteronuclear multiple bond correlation (HMBC) analyses.

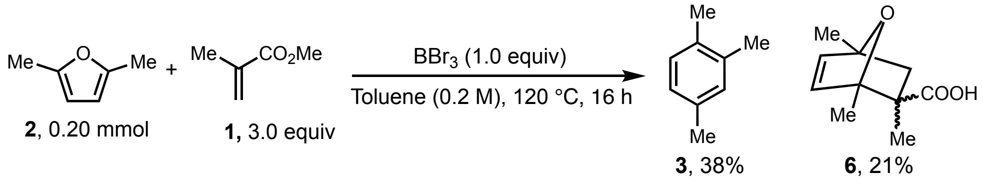

exo- and *endo*-**6** mixture: HRMS (ESI-TOF, CH_3OH) m/z: $[M + Na]^+$ calculated for $C_{10}H_{14}O_3Na$ 205.0841, found 205.0834.

endo-**6** (minor): ^{1}H NMR (500 MHz, $CDCl_3$) δ 5.80 (dq, *J* = 5.6, 1.7 Hz, 1H, ^{h}H), 4.32 (d, *J* = 5.6 Hz, 1H, ^{i}H), 2.24 (d, *J* = 18.8 Hz, 1H, $^{j/k}$H), 2.15 (d, *J* = 18.8 Hz, 1H, $^{j/k}$H), 1.77 (s, 3H, $^{l/m/n}$H), 1.38 (s, 3H, $^{l/m/n}$H), 1.14 (s, 3H, $^{l/m/n}$H).

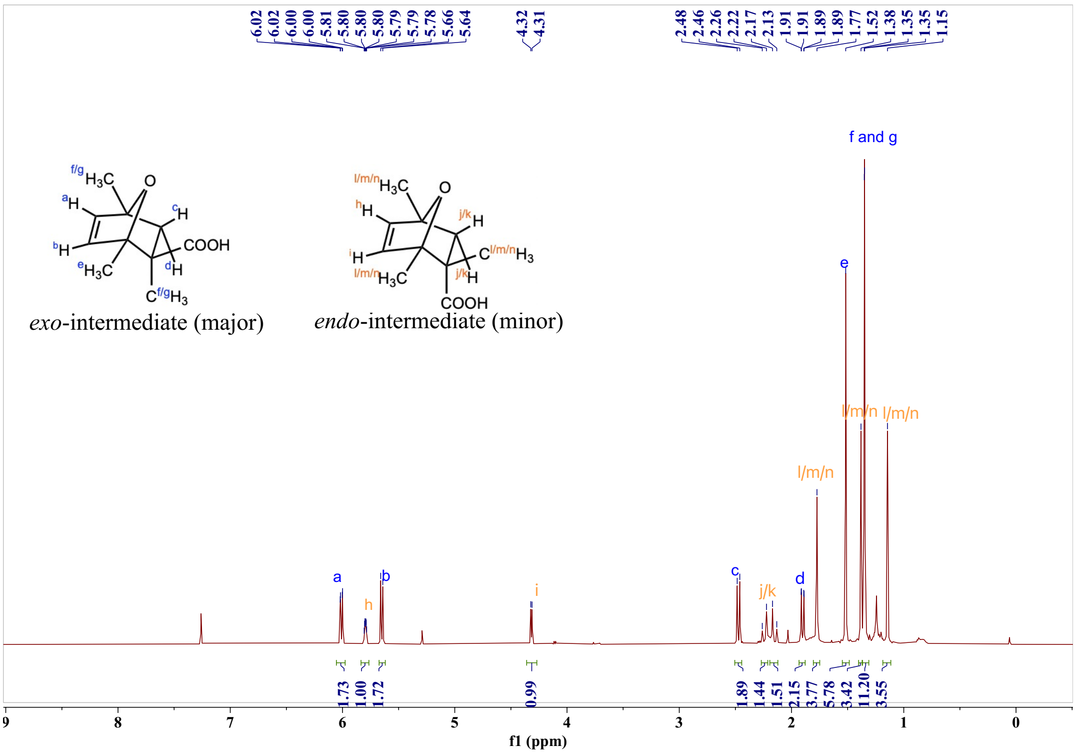

Figure S12. ^{1}H-NMR (500 MHz, $CDCl_3$) spectrum of mixture of *endo*-**6** and *exo*-**6** in ratio of 1.00:1.73

exo- and *endo*-**6** mixture: ^{13}C NMR (126 MHz, $CDCl_3$) δ 179.7, 179.5, 144.0, 136.1, 134.9, 120.4, 80.0, 78.7, 75.4, 70.6, 52.8, 47.5, 46.6, 39.3, 24.2, 23.1, 22.0, 20.6, 16.0, 14.5.

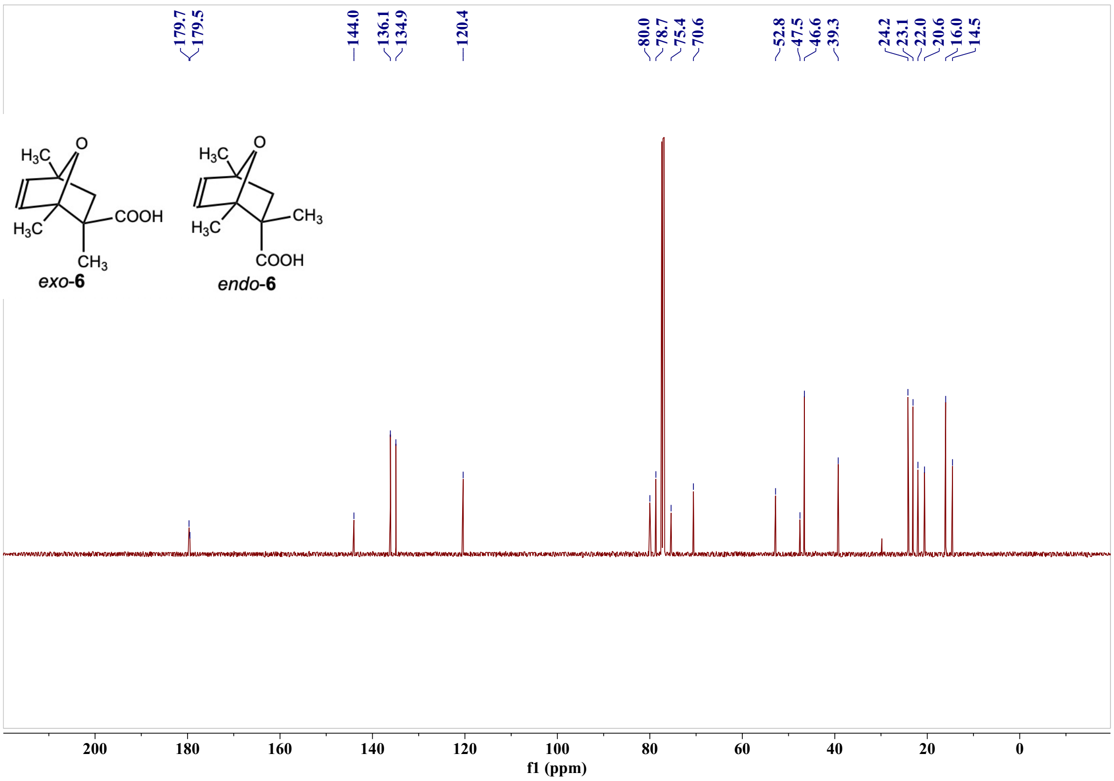

Figure S13 $^{13}C\{^1H\}$-NMR (126 MHz, $CDCl_3$) spectrum of mixture of *endo*-**6** and *exo*-**6**.

exo-**6** (major): ^{1}H NMR (500 MHz, $CDCl_3$) δ 6.03 (dd, *J* = 9.3, 1.3 Hz, 1H, ^{a}H), 5.66 (d, *J* = 9.3 Hz, 1H, ^{b}H), 2.47 (d, *J* = 11.6 Hz, 1H, ^{c}H), 1.92 (dd, *J* = 11.6, 1.3 Hz, 1H, ^{d}H), 1.53 (s, 3H, ^{e}H), 1.38 – 1.34 (m, 6H, ^{f}H and ^{g}H).

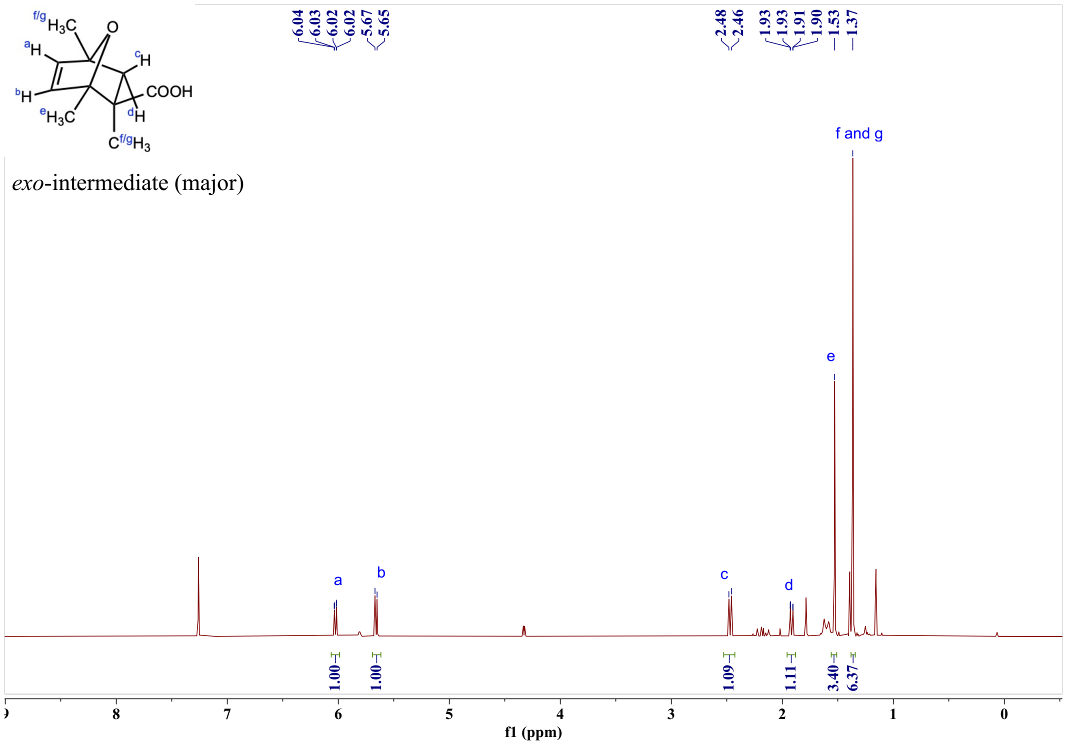

Figure S14. ^{1}H-NMR (500 MHz, $CDCl_3$) spectrum of *exo*-**6**.

exo-**6** (major): ^{13}C NMR (126 MHz, $CDCl_3$) δ 179.6 ([a]C, COOH), 136.1([b]C, alkene), 135.0 ([c]C, alkene), 79.9 ([d]C, quaternary, connected to bridge O), 70.7 ([e]C, quaternary, connected to bridge O), 52.8 ([f]C, quaternary, connected to COOH), 46.6 ([g]C, secondary), 24.2 ([h]C, primary), 23.1 ([i]C, primary), 16.0 ([j]C, primary).

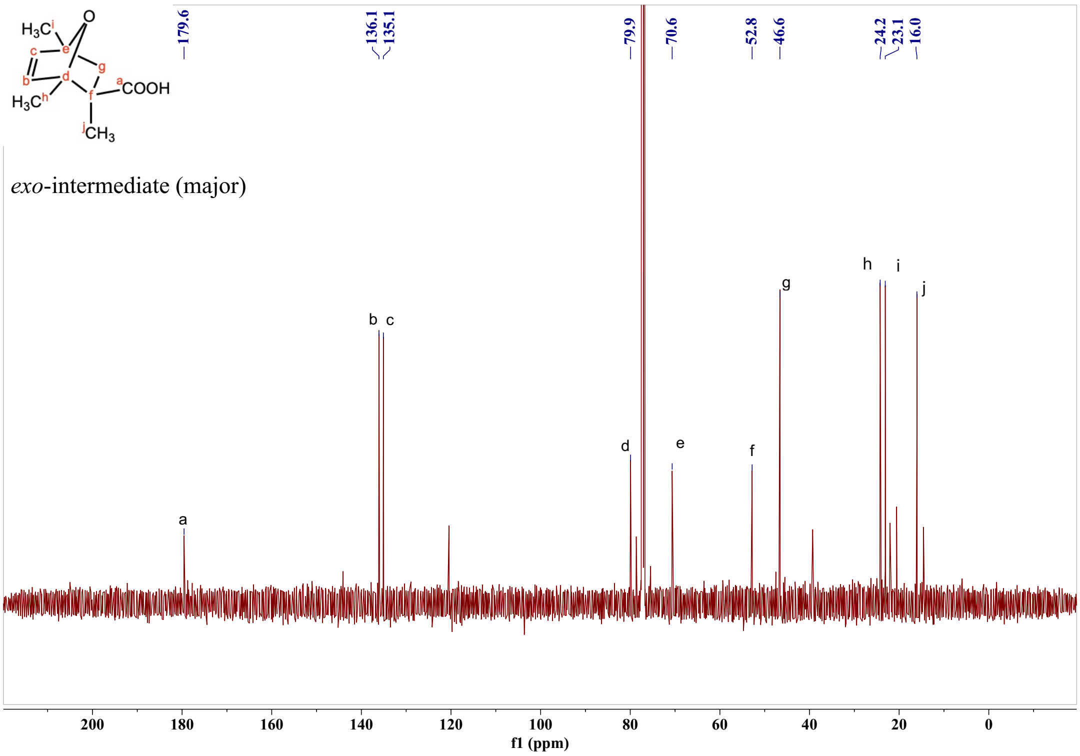

Figure S15 $^{13}C\{^1H\}$-NMR (126 MHz, $CDCl_3$) spectrum of *exo*-**6**.

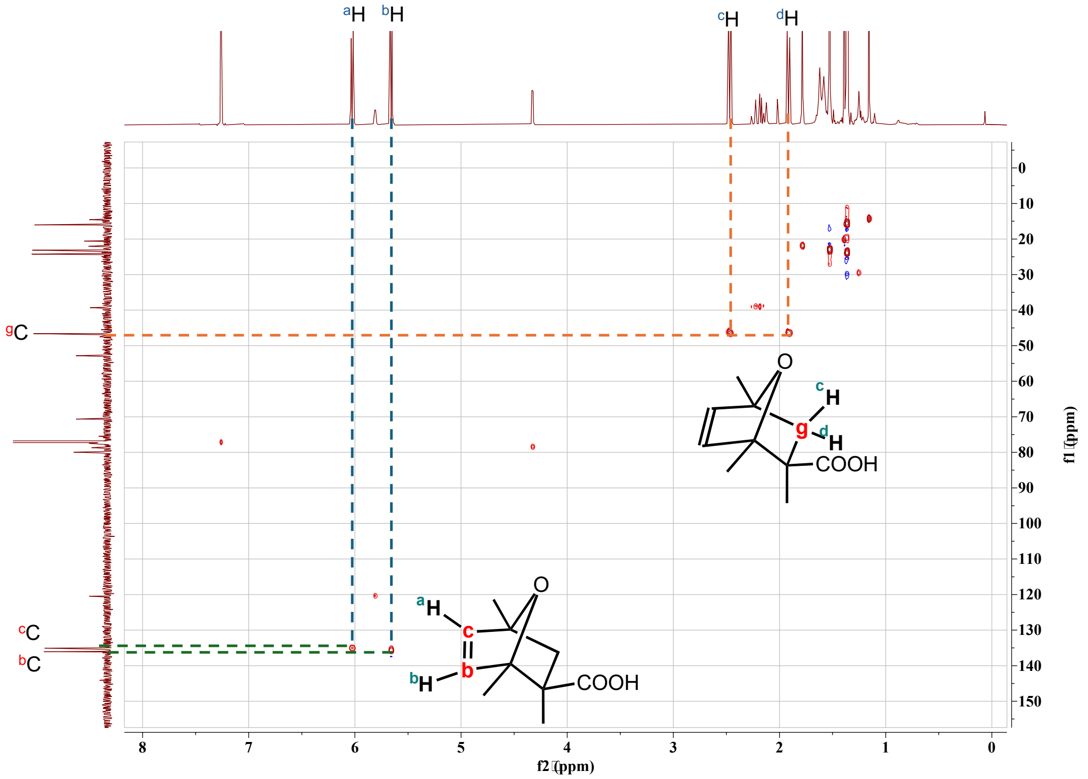

Figure S16 $^{1}H/^{13}C\{^{1}H\}$-HSQC NMR (500 MHz, $CDCl_3$) spectrum of *exo*-**6**.

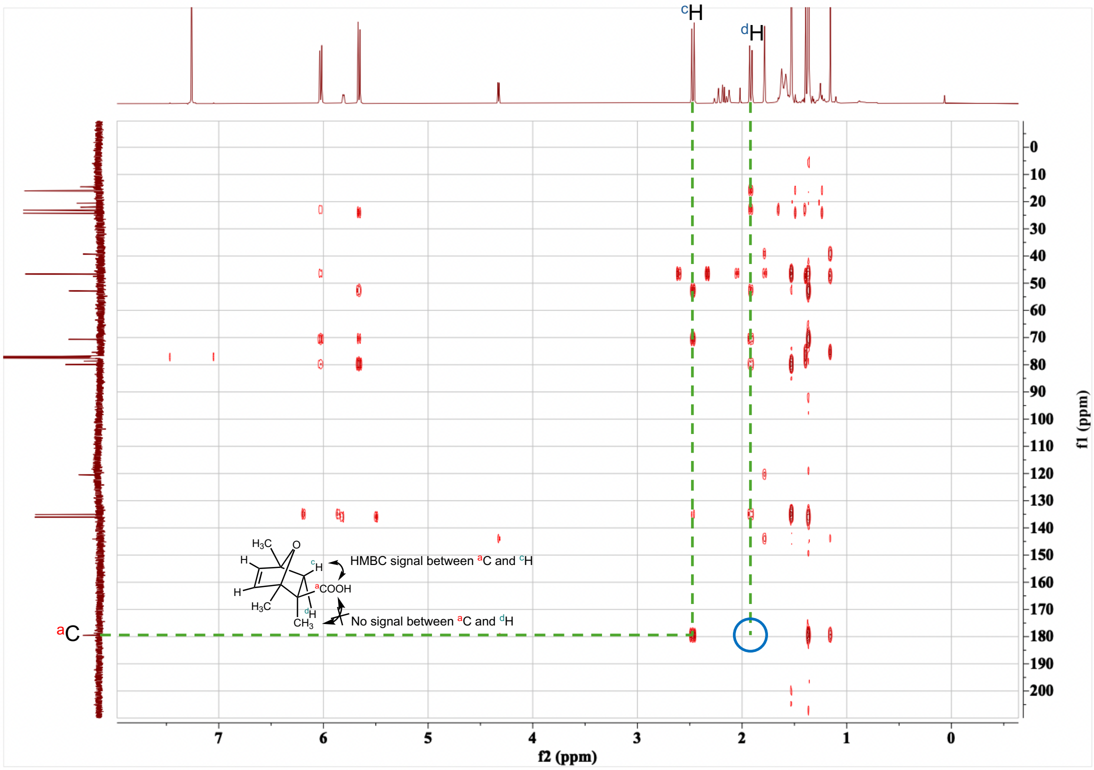

Figure S17 $^{1}H/^{13}C\{^{1}H\}$-HMBC NMR (500 MHz, $CDCl_3$) spectrum of *exo*-**6**.
